# Editorial: Manipulation of the cellular microbicidal response and endocytic dynamic by pathogens membrane factors

**DOI:** 10.3389/fcimb.2015.00042

**Published:** 2015-05-29

**Authors:** Benjamin Coiffard, Philippe Soubeyran, Eric Ghigo

**Affiliations:** ^1^Department of Pulmonology and Lung Transplantation, Hôpital NordMarseille, France; ^2^Cellular Stress, Centre de Recherche en Cancérologie de Marseille, Institut National de la Santé et de la Recherche Médicale UMR 1068, Centre National de la Recherche Scientifique UMR 7258, Institut Paoli-CalmettesMarseille, France; ^3^Faculté de Médecine, URMITE-IRD198, Centre National de la Recherche Scientifique UMR7278Marseille, France

**Keywords:** planarians, coxiella, body lices, granulomas, ubiquitination, host-pathogens interaction, mycobacteria

Microbes such as bacteria, parasites and fungi, have evolved specialized mechanisms to survive and replicate in their host. While high pathogen reproduction is the main purpose to improve next generation growth, adaptation of pathogens to their hosts depends on factors affecting mostly their survival rate. The principle of these mechanisms is to hijack the microbicidal cell function in order to disable and destabilize the host cell defense that. controls and eliminates foreign invaders. Devoid of their defense, cells become permissive to pathogens invasion, a phenomenon that leads to disorders and diseases. To counterstrike the microbicidal functions of the host, microbes use a large arsenal of molecules, known as virulence factors, ranging from proteins and lipids to saccharides. Several evidences highlighted that pathogens use these molecules in order to interfere with the phagolysosome biogenesis, to reprogram signal transduction pathways and, therefore, create a replicative niche.

This special issue covers recent understanding of mechanisms and molecules used by bacterial pathogens such as *Coxiella burnetii* (LPS), *Mycobacterium tuberculosis* (LAM) and parasites such as Leishmania (LPG) to interfere with the microbicidal function of cells (e.g., Rab network, ubiquinilation, TLRs signaling). Attention is mainly focused on the reprogramming of the cellular dynamics (granulomas formation), immune response, phagolysosome biogenesis and signal transduction pathways by pathogens. Thus, Vergne and colleagues well summarize the scientific literature on Lipoarabinomannan, a major immunomodulatory lipoglycan found in the cell envelope of *Mycobacterium tuberculosis*, focusing their attention on its structure and its ability to manipulate the endocytic pathway as well as phagocyte functions (Vergne et al., 2015). In similar manner, the review of Astarie-Dequeker group, address exclusively the role played by phthiocerol dimycocerosates in the modulation of the resident macrophage response (Arbues et al., 2014). Similarly, the composition of the *Leishmania* lipophosphoglycan, its peculiar chemical structure and what is currently known about its effects favoring parasite virulence in the mammalian host, are the subject of a short perspective written by Forestier et al. (2015). The capacity for bacteria to used LPS to hijack molecular process is highlighted by Conti and colleagues which described how *C. burnetii* avoids macrophage activation by the disruption of the TLR-2 and TLR-4 association through the reorganization of the macrophage cytoskeleton by *C. burnetii* LPS (Conti et al., 2015); the same pathogen is the object of the work of Faugaret which have studied the molecular mechanisms of granuloma formation in response to *C. burnetii* and found that it is that it is associated with a core of transcriptional response based on inflammatory genes (Faugaret et al., 2014). Finally, Mottola comments and emphasizes the recent discoveries on bacterial pathogens that control the localization or function of the small GTPases Rab5 and Rab7, and therefore modify the maturation from early to late phagosomes (Mottola, 2014). Alomairi and colleagues recapitulate what is currently known about the normal functions of ubiquitination during host cell infection, and they highlight its hijacking to escape clearance and proliferate (Alomairi et al., 2015). It is also important to note that model organisms in the continuous effort to decipher the role of the molecular players involved, contribute strongly to the study of host pathogen interaction and to the discovery of new virulence factors or microbicidal mechanisms: in this special issue, three articles will discuss their invaluable characteristics, with a special attention to the unconventional animal models called also exotic models or ExoMod (Conti et al., 2014; Abnave et al., 2015; Coulaud et al., 2015).

The issue of drug resistance is as old as antibiotics themselves, but so far very few steps have been undertaken to reduce the impact of this threatening public health menace. Beyond the variety of novel approaches being utilized by biotech companies, fundamental research is essential to elucidate how microbes replicate in the host, how molecular players are involved in the host-parasite interactions and how intracellular pathogens finally could become resistant to drugs. Conversely, the capacity of pathogens to perturb the microbicial response can be used to define new therapeutic strategies, and as tools to investigate cells properties: Gorvel and colleagues exploited *C. burnetii* and *Brucella abortus* as tools to elucidate the role of a specific subpopulations of Dendritic cells, the decidual Dendritic cells (dDCs), in placental immune system (Gorvel et al., 2015).

## Conflict of interest statement

The authors declare that the research was conducted in the absence of any commercial or financial relationships that could be construed as a potential conflict of interest.
